# Genetic Analysis of *S5*-Interacting Genes Regulating Hybrid Sterility in Rice

**DOI:** 10.1186/s12284-020-00452-x

**Published:** 2021-01-09

**Authors:** Jianglei Rao, Xing Wang, Zhongquan Cai, Yourong Fan, Jiangyi Yang

**Affiliations:** State Key Laboratory for Conservation and Utilization of Subtropical Agro-Bioresources, College of Life Science and Technology, Guangxi University, Nanning, 530004 China

**Keywords:** Hybrid incompatibility, Wide compatibility, Reproductive isolation, Spikelet fertility, Quantitative trait loci mapping

## Abstract

**Background:**

Asian cultivated rice (*Oryza sativa* L.) comprises two subspecies, *O. sativa* subsp. *indica* and subsp. *japonica*, and the hybrids between them display strong heterosis. However, hybrid sterility (HS) limits practical use of the heterosis between these two subspecies. *S5* is a major-effect locus controlling the HS of female gametes in rice, consisting of three closely-linked genes *ORF3*, *ORF4* and *ORF5* that act as a killer-protector system. The HS effects of *S5* are inconsistent for different genetic backgrounds, indicating the existence of interacting genes within the genome.

**Results:**

In the present study, the *S5*-interacting genes (SIG) and their effects on HS were analyzed by studying the hybrid progeny between an *indica* rice, Dular (DL) and a *japonica* rice, Balilla^*ORF5*+^ (BL^*ORF5*+^), with a transgenic *ORF5+* allele. Four interacting quantitative trait loci (QTL): *qSIG3.1*, *qSIG3.2*, *qSIG6.1*, and *qSIG12.1*, were genetically mapped. To analyze the effect of each interacting locus, four near-isogenic lines (NILs) were developed. The effect of each specific locus was investigated while the other three loci were kept DL homozygous (DL/DL). Of the four loci, *qSIG3.1* was the SIG with the greatest effects in which the DL allele was completely dominant. Furthermore, the DL allele displayed incomplete dominance at *qSIG3.2*, *qSIG6.1*, and *qSIG12.1*. *qSIG3.1* will be the first choice for further fine-mapping.

**Conclusions:**

Four *S5*-interacting QTL were identified by genetic mapping and the effect of each locus was analyzed using advanced backcrossed NILs. The present study will facilitate elucidation of the molecular mechanism of rice HS caused by *S5*. Additionally, it would provide the basis to explore the origin and differentiation of cultivated rice, having practical significance for inter-subspecific hybrid rice breeding programs.

**Supplementary Information:**

The online version contains supplementary material available at 10.1186/s12284-020-00452-x.

## Background

Reproductive isolation provides the impetus for the formation and maintenance of species due to a reduction in gene flow between species or subspecies (Oka 1988). Depending on the developmental stage, reproductive isolation can be categorized as prezygotic and postzygotic ones (Seehausen et al. 2014). Prezygotic reproductive isolation prevents the formation of hybrid zygotes, while postzygotic reproductive isolation can lead to hybrid incompatibility (Ouyang and Zhang 2013). Inter-specific or inter-subspecific hybrid sterility (HS) is a common phenomenon causing postzygotic reproductive isolation (Ouyang and Zhang 2013). In addition to HS, postzygotic reproductive isolation can also result in hybrid lethality or weakness in F_1_ plants or their offspring (Ouyang and Zhang 2013; Fishman and Sweigart 2018).

As a staple food crop, sustainable production of high-yield rice is essential for global food security (Peng et al. 2008; Khush and Gupta 2013). Asian cultivated rice (*Oryza sativa* L.) comprises two subspecies: *indica* and *japonica*, and hybrids of the two display strong heterosis (Yuan 1994; Zhang et al. 1996; Zhao et al. 1999). However, the value of inter-subspecific heterosis is somewhat limited due to HS (Ikehashi et al. 1994; Liu et al. 1996). Kato et al. (1928) demonstrated that the fertility of *indica-japonica* hybrid F_1_ plants ranged from 0% to 33%. Subsequently, a large number of HS loci were identified in rice (Ouyang et al. 2009). Of these, 11 HS genes have been cloned. The majority of cloned genes are related to the hybrid pollen sterility, such as *Sa*, *DPL1/DPL2*, *S27/S28*, *Sc*, *DGS1/DGS2* and *qHMS7* (Long et al. 2008; Mizuta et al. 2010; Yamagata et al. 2010; Nguyen et al. 2017; Shen et al. 2017; Yu et al. 2018). Several are related to hybrid embryo sac sterility, such as *S5*, *hsa1*, *S7*, and *ESA1* (Yang et al. 2012; Kubo et al. 2016; Yu et al. 2016; Hou et al. 2019). Interestingly, *S1* controls both male and female hybrid sterility (Xie et al. 2017; Koide et al. 2018; Xie et al. 2019).

A kind of special rice was called wide compatibility varieties (WCVs), whose hybrids display normal fertility when crossed with *indica* or *japonica* rice (Ikehashi and Araki 1984). Therefore, utilization of WCVs is considered a method of overcoming HS in rice. Dular, an *indica* rice from India, is a typical WCV (Liu et al. 1996; Zhang et al. 1997). The first wide compatibility gene (WCG), *S5*, has been identified as a major-effect locus controlling HS and wide compatibility (Ikehashi and Araki 1986; Liu et al. 1992; Liu et al. 1997; Wang et al. 1998; Qiu et al. 2005; Song et al. 2005; Chen et al. 2008), which is also relevant to the evolutionary origin and differentiation of rice (Du et al. 2011; Ouyang et al. 2016; Mi et al. 2020). Initial research inferred that there are three alleles at the *S5* locus: *S5*-*i* (*indica* rice), *S5*-*j* (*japonica* rice), and *S5*-*n* (WCVs). The fertility of hybrids containing *S5*-*n*/*S5*-*i* and *S5*-*n*/*S5*-*j* are normal, while those containing *S5*-*i*/*S5*-*j* are semi-sterile (Ikehashi and Araki 1986). A later study demonstrated that the *S5* locus consists of three closely linked genes (*ORF3*, *ORF4* and *ORF5*), which form a killer-protector system that regulates hybrid fertility (Yang et al. 2012). Both *ORF4* and *ORF5* have sporophytic mode of action, while *ORF3* has gametophytic one. Typical *japonica* varieties carry *ORF3−*/*ORF4+*/*ORF5-* haplotype, while typical *indica* varieties carry *ORF3+*/*ORF4−*/*ORF5+* haplotype (here “+” represents functional allele and “**-**” represents non-functional allele). In *indica*/*japonica* hybrids, *ORF5+* and *ORF4+* synergistically abort unguarded female gametes (carrying *ORF3-*, usually *japonica* gametes), while guarded female gametes (carrying *ORF3+*, usually *indica* gametes) is still live (Yang et al. 2012). Transcriptome analysis has shown that the ORF5+ protein might destroy the integrity of the cell wall, and signals are transmitted by transmembrane protein ORF4+ into the cell, resulting in severe endoplasmic reticulum stress, eventually leading to female gamete abortion (Yang et al. 2012; Zhu et al. 2017). Nevertheless, during this process, ORF3+, an Hsp70 protein, can prevent endoplasmic reticulum stress and allow the production of fertile gametes (Yang et al. 2012; Zhu et al. 2017).

It was observed that the effect of *S5* varies depending upon the genetic background, indicating that unknown background gene(s) control HS by interacting with *S5* (Yang 2012). The aim of the present study was to map those *S5*-interacting genes (SIGs), and develop near-isogenic lines (NILs) to analyze and verify the effects of each SIG, laying the foundation for genetic fine-mapping of SIG in the future.

## Methods

### Genetic Material

**Fig. 1:**
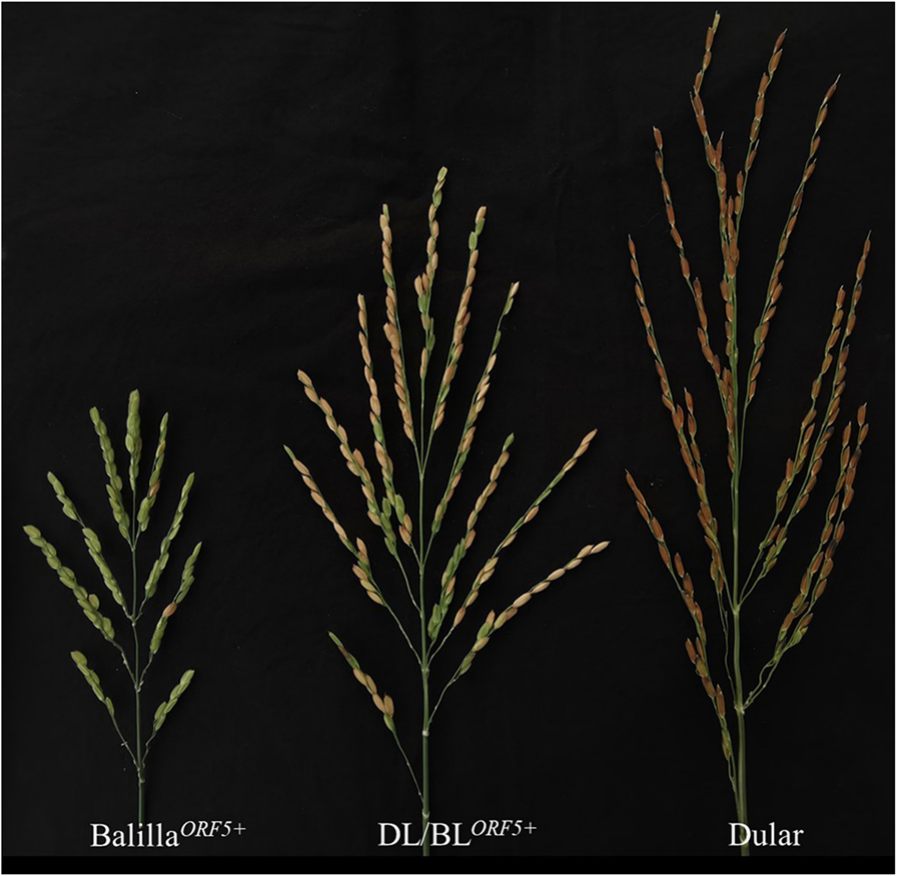
Spikelet fertility of Balilla^*ORF5+*^, Dular and DL/BL^*ORF5+*^. The spikelet fertility of male parent Balilla^*ORF5+*^ was < 5%, while the female Dular parent exhibited normal spikelet fertility. Spikelet fertility of the hybrid F_1_ (DL/BL^*ORF5+*^) was approximately 65%. DL, Dular; BL, Balilla

Balilla (abbreviated as BL, with *S5* haplotype *ORF3−*/*ORF4+*/*ORF5-*) is a temperate *japonica* rice introduced from Italy. BL^*ORF5+*^ was obtained by transforming the *ORF5+* allele of the *S5* locus into BL (Chen et al. 2008) (Fig. S1a). Positive transgenic plants BL^*ORF5+*^ possess the suicidal *S5* genotype (*ORF3-*, *ORF4+*, and *ORF5+*, “+” representing the functional allele, “**-**” representing the non-functional allele), that displayed extremely low spikelet fertility (SF) (Fig. 1; Table 1). Dular (abbreviated as DL, with *S5* haplotype *ORF3−*/*ORF4−*/*ORF5n*, “n” representing a non-functional allele) is an *indica*-type WCV introduced from India (Fig. 1; Table 1). F_1_ plants DL/BL^*ORF5+*^ with the suicidal *S5* genotype were derived from a cross between DL and BL^*ORF5+*^ (Fig. 1; Table 1). A total of 173 individual plants (*ORF5+* transgenic positive) of the F_2_ population were selected for QTL mapping. Other populations, such as BC_3_F_3_, BC_4_F_6_, and BC_6_F_4_ were developed by phenotypic selection and molecular marker-assisted selection, with BL as the recurrent parent. All plants were planted in the experimental fields of Guangxi University, Nanning, China.

**Table 1:** Genotype and SF of rice materials

Material	*S5* genotype	SF (%)
BL	*ORF3-*, *ORF4+*, *ORF5-*	> 80
DL	*ORF3-*, *ORF4-*, *ORF5n*	> 80
BL^*ORF5+*^	*ORF3-*, *ORF4+*, *ORF5−*/^*ORF5+*^	< 5
DL/BL^*ORF5+*^	*ORF3-*, *ORF4****+***/*ORF4-*, *ORF5−*/*ORF5n*/^*ORF5+*^	> 65

*SF* spikelet fertility, *BL* Balilla, *DL* Dular, *BL*^*ORF5+*^ Balilla^*ORF5+*^, ^*ORF5+*^, transgenic *ORF5+* allele. For homozygotes, only one allele is shown. For heterozygotes, only the functional allele is shown in the text

### Development of NILs to Analyze the Effect of each QTL

Before the related QTLs were determined, all transgenic plants with both suicidal *S5* genotype and higher SF were selected to backcross with BL. The recurrent female parent BL has the *S5* haplotype *ORF3−*/*ORF4+*/*ORF5-*, and the introduction of transgenic *ORF5+* results in a suicidal *S5* genotype (*ORF3-*, *ORF4+*, and *ORF5+*). To isolate those plants carrying the suicidal *S5* genotype (*ORF3-*, *ORF4+*, and *ORF5+*), it was necessary to first identify the transgenic *ORF5+* gene. Insertion and deletion (InDel) marker S5P50 (primer sequences detailed in Table S1), in the promoter region of *ORF5+*, has already been developed (Yang et al. 2012) (Fig. S1b). For further confirmation, histological staining was employed for reporter gene GUS (*β-glucuronidase*) to detect transgenic plants (Fig. S1c), which stained a blue color (Fig. S1c).

After the related QTLs had been determined, backcrossing could be facilitated by marker-assisted selection. Finally, selfing progeny of single-locus heterozygous (the other three loci were DL/DL homozygous, transgene *ORF5+* was hemizygous) were used to analyze the effect of each QTL. For simplification, single-locus separating NILs for *qSIG3.1*, *qSIG3.2*, *qSIG6.1* and *qSIG12.1* were termed *qSIG3.1-*NIL, *qSIG3.2-*NIL, *qSIG6.1-*NIL and *qSIG12.1-*NIL, respectively (Table S2).

### DNA Extraction and Genotyping

Total DNA was extracted from 1 g fresh rice leaves using cetyl trimethyl ammonium bromide (CTAB) (Murray and Thompson 1980). Whole-genome InDel markers based on PCR were designed using rice genome sequence data (http://ricevarmap.ncpgr.cn/v2/). The names of markers defined their physical location on the respective chromosome. For example, marker C014115 was at 4115 kb on chromosome 1. Finally, 169 polymorphic InDel markers were selected for QTL mapping.

The InDel marker S5P50 (Yang et al. 2012) and GUS staining were used to identify transgenic *ORF5+* plants. The positive plants were further genotyped using three primers: TL, TRB, and TR (Table S1) to detect copy numbers of foreign *ORF5+* fragments. The three primers were designed using flank sequences of transgenic foreign *ORF5+* fragment insertion. Additionally, the genotypes of BC_6_F_2_ generation plants were detected using a rice genome 6 K SNP microarray (China National Seed Group Co., Ltd).

### Investigation of SF and Data Analysis

Three to five panicles were harvested from each individual plant to calculate the mean SF value. SF was measured as the proportion of well-developed fertile spikelets over the total number of spikelets. Mean values, standard deviations (SD), standard error of the mean (SEM), and significance of differences were evaluated using SPSS v17.0 software (SPSS Inc., United States). The significance of differences was calculated using a *t*-test.

### Construction of Genetic Linkage Map and QTL Mapping

The construction of genetic map and QTL mapping were conducted using IciMapping 4.0 software (www.isbreeding.net). After genotype data were imported into the software, markers were grouped only by anchor, ordered using the nnTwoOpt algorithm (nearest neighbor was used for tour construction, and two-opt was used for tour improvement) and rippled by the sum of adjacent recommendation frequencies (SARF) (Li et al. 2008). The Kosambi mapping function was used to estimate the genetic distance between markers. QTL were analyzed by inclusive composite interval mapping (ICIM) where LOD = 3.

### Isolation of Transgenic *ORF5+* Flanking Sequences and Development of a DNA Marker

The total genomic DNA of *ORF5+* transgene-positive plants was cleaved using a single restriction endonuclease enzyme from multiple cloning sites of the *ORF5+* transformation vector (pCAMBIA1301) (Chen et al. 2008). Foreign transgenic DNA fragments and the flanking genome DNA fragments then self-ligated into rings using T4 DNA ligase. Inverse PCR was performed to amplify the flanking sequence, using primer pair ULB2 and pCM13-L or XRB1, and pCM13-R, the sequences of which are detailed in Table S1. In the present study, clear bands were observed in the PCR products after digestion with *Bam*H I or *Xba* I and amplification with ULB2 and pCM13-L. The PCR products were then sequenced and matched with the rice genome database to verify the location of the transgenic insertion.

For the next step, the three primers TL, TRB and TR, were designed to detect whether the transgenic insertion of *ORF5+* was homozygous or hemizygous. Primers TL and TR were designed using the rice genome sequence and matched to the left and right side of the insertion position, respectively. The remaining primer, TRB was designed to match the components of the transgenic vector (Fig. S1d). If there was an *ORF5+* transgenic insertion, a 170-bp fragment would be amplified using primers TRB and TR. Otherwise, a 200-bp fragment was amplified with primers TL and TR (Fig. S1e). Therefore, the genotypes of transgenic *ORF5+* plants could easily be distinguished: one band at 200-bp for transgenic *ORF5+* negative plants, two bands at 170-bp and 200-bp for the hemizygous *ORF5+* transgenic plants, and only one band at 170-bp for the homozygous *ORF5+* transgenic plants (Fig. S1e). The PCR products were examined using 2.5% agarose gel electrophoresis.

## Results

In the present study, QTL scanning and mapping of *qSIG3.1* and *qSIG5.1* were conducted using an F_2_ population. Due to the large effects of *qSIG3.1* and *qSIG5.1*, other loci could not be identified in the F_2_ population. Surprisingly, a single *qSIG3.1* locus cannot reflect its due effect in backcrossed progeny. Using marker-assisted backcrossing supplemented with phenotypic selection, we found *qSIG3.2* and *qSIG12.1* in the BC_3_F_3_ population via genome-wide molecular marker detection. In the BC_6_F_2_ generation, two plants did not show the expected SF. A rice genome 6 K SNP microarray was used to detect these two plants and found *qSIG6.1*. These QTL were detected by different methods in different populations.

### Phenotypic Data of Parents and F_1_ Plants

The SF of the male parent BL^*ORF5+*^ was < 5%, while that of female parent DL was > 80% (Fig. 1). The DL/BL^*ORF5+*^ F_1_ plants were derived from a cross between DL and BL^*ORF5+*^, whose SF was as high as 65% (Fig. 1), irrespective of its suicidal *S5* genotype (Table 1). Since both BL^*ORF5+*^ and DL/BL^*ORF5+*^ possessed the same suicidal *S5* genotype, the substantial SF differences between them indicated the existence of *S5*-interacting genes in the background genome.

### Construction of Genetic Linkage Map and QTL Mapping in the F_2_ Population

**Fig. 2:**
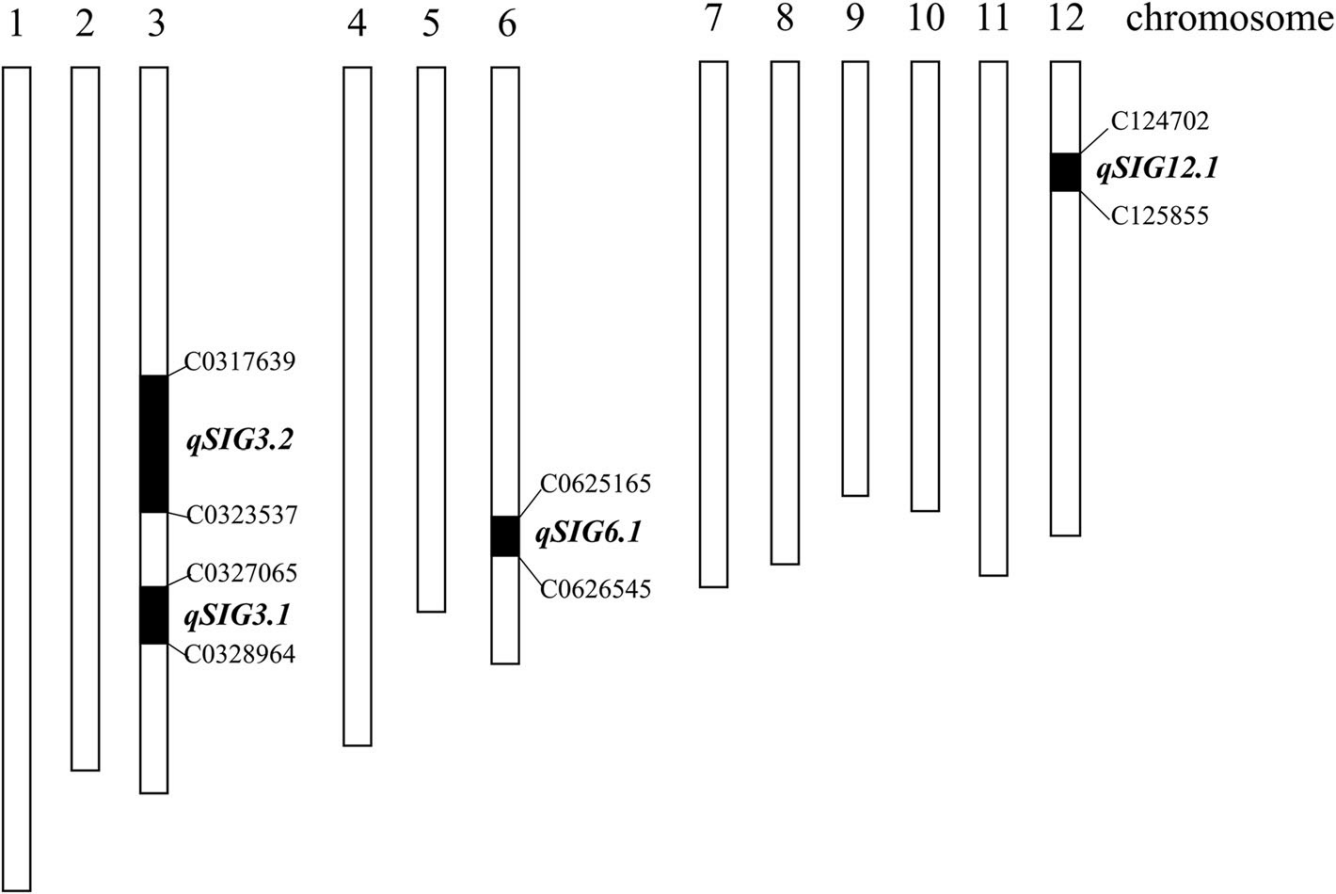
Location of four QTLs on chromosomes. Markers were named after their physical location on the chromosome. For example, C0317639 indicates that the marker is at 17,639 kb on chromosome 3

In the F_2_ population of DL/BL^*ORF5+*^, 173 individuals carrying the suicidal *S5* genotype (*ORF3-*, *ORF4+*, and *ORF5+*) were genotyped using 169 InDel markers. A genetic map was constructed with IciMapping 4.0 software, covering 1552.45 cM, with a mean distance of 9.19 cM between markers (Fig. S2). Two QTL *qSIG3.1* and *qSIG5.1* were mapped, using inclusive composite interval mapping with LOD ≥3. *qSIG3.1* was located on chromosome 3, between markers C0326556 and C0328430 with a LOD of 3.18. The rate of phenotypic variation explained (PVE) by *qSIG3.1* was 7.02% (Table 2). The additive (Add) and dominance (Dom) effects of *qSIG3.1* were − 0.049 and 0.0763, respectively (Table 2). QTL *qSIG5.1* was located on chromosome 5, between markers C051419 and C055412 with a LOD of 3.70 and PVE of 10.44% (Table 2). The Add and Dom of *qSIG5.1* were − 0.038 and 0.0957, respectively (Table 2). In subsequent analysis, the location of *qSIG3.1* was narrowed down to between markers C0327065 and C0328964 (Fig. 2) using several recombinants in BC_4_F_3_ (Fig. S3).

**Table 2:** Details of QTLs mapped in the F_2_ population

QTL	Position	Chr	Left Marker	Right Marker	LOD	PVE (%)	Add	Dom
*qSIG3.1*	138	3	C0326556	C0328430	3.18	7.02	−0.049	0.0763
*qSIG5.1*	33	5	C051419	C055412	3.7	10.44	−0.038	0.0957

*QTL* quantitative trait loci, *Chr* chromosome, *LOD* log odds score, *PVE* phenotypic variation explained by the marker, *Add* additive effect, *Dom* dominance effect. Markers were named after their physical location on the chromosome. For example, C0326556 indicates that the marker is at 26,556 kb on chromosome 3

### QTL *qSIG5.1* Is the Insertion Site of *ORF5+*

In the F_2_ population, we observed severe segregation distortion at *qSIG5.1*. Of the 173 F_2_ individuals, only two were genotyped as DL/DL homozygous at the right flank marker C055412 (Table S3). Since all 173 F_2_ plants originated from the same transgenic *ORF5+* plant, the foreign *ORF5+* transgene should have been inserted into the same site of the BL genome. The closely linked markers of *ORF5+* insertion site should be BL/BL homozygous or BL/DL heterozygous genotypes, with no DL/DL homozygotes. This deduction could well explain the serious segregation distortion at *qSIG5.1*. So we speculated that *qSIG5.1* was in fact the insertion site of *ORF5+*.

In order to confirm this speculation, we isolated the flanking sequence of the insertion site of transgenic *ORF5+*. The foreign *ORF5+* transgene was inserted between 4,621,102 bp and 4,621,103 bp on chromosome 5, just within the region of *qSIG5.1*. From the sequence information of the insertion position, three primers TL, TRB, and TR were designed to assess whether the transgenic *ORF5+* was a hemizygote or homozygote (Fig. S1d).

### Mapping *qSIG3.2* and *qSIG12.1* in the Backcrossed Progeny Using InDel Markers

In the backcrossed BC_3_ populations of *qSIG3.1*, an interesting observation was made. In a number of families, the SF of all the plants was less than 15%, irrespective of the *qSIG3.1* genotype. In other families, the SF of plants with the same *qSIG3.1* genotype varied widely. We speculated that other loci were affecting the SF of the plants. Two BC_3_F_3_ families with different SF were identified. The individuals with high SF (> 35%) were selected and genotyped with whole-genome InDel markers. Each plant carried two additional fragments from the DL variety (Fig. S4). One, named *qSIG3.2*, was narrowed down to be between markers C0317639 and C0323537, while a second, named *qSIG12.1*, was located between markers C124702 and C125855 (Fig. 2).

### Mapping of *qSIG6.1* in the Backcrossed Progeny Using an SNP Microarray

In a BC_6_F_2_ family, the genotypes of two plants, 18MR47–17 and 18MR47–19, were found to be similar. Both were BL/DL at *qSIG3.2* and DL/DL at *qSIG12.1*, but 18MR47–17 and 18MR47–19 were BL/DL and DL/DL at *qSIG3.1* respectively. Additional investigation revealed that the DL allele at *qSIG3.1* was able to increase SF (see the section below about genetic effects analysis of *qSIG3.1*), and the DL allele at *qSIG3.1* was completely dominant. Therefore, the SF of these two plants should be identical. However, the SF of 18MR47–17 was 44.43 ± 6.75%, and that of 18MR47–19 was only 19.79 ± 4.81% (Table 3), indicating the presence of other interacting loci.

**Table 3:** Genotype and SF of plants 18MR47–19 and 18MR47–17

Generation	Plant No.	QTL Genotype			*ORF5+*	SF (%) ^a^
		*qSIG3.1*	*qSIG3.2*	*qSIG12.1*		
BC_6_F_2_	18MR47–19	DL/DL	BL/DL	DL/DL	hemizygote	19.79 ± 4.81
BC_6_F_2_	18MR47–17	BL/DL	BL/DL	DL/DL	hemizygote	44.43 ± 6.75

*SF* spikelet fertility, *BL/DL* heterozygous Balilla/Dular, *DL/DL* homozygous Dular/Dular. ^a^means ± SD

To identify the new interacting loci, the genomes of these two individual plants were analyzed using a rice genome 6 K SNP microarray and the results were compared with their recurrent parent, BL. The microarray results indicated that 18MR47–17 and 18MR47–19 had different genotypes on chromosomes 6 and 12 (Fig. S5). 18MR47–17 which exhibited a higher SF was heterozygous BL/DL in both regions. On the other hand, 18MR47–19, with a lower SF, carried BL/BL and DL/DL on chromosomes 6 and 12 respectively. We speculated that the DL segment of chromosome 6 was able to increase the fertility of plant 18MR47–17. We termed this locus *qSIG6.1*. Based on the SNP information from the rice genome 6 K SNP microarray, a number of additional InDel markers were developed to verify the genotypes of 18MR47–17 and 18MR47–19 (Table S1). The location of QTL *qSIG6.1* was finally identified as between markers C0625165 and C0626545 (Fig. 2).

### Genetic Effects Analysis of qSIG3.1, qSIG3.2, qSIG6.1, qSIG12.1, and ORF5+

**Fig. 3:**
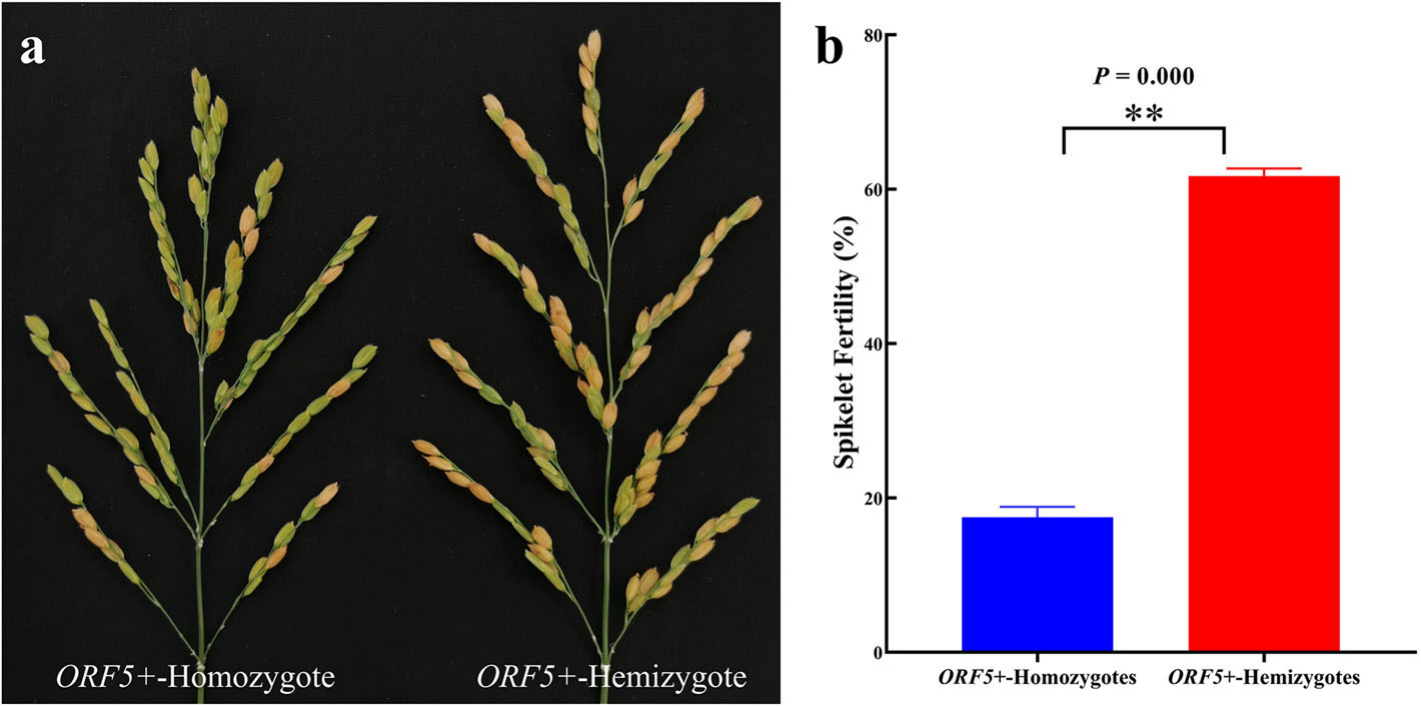
Effect of transgene *ORF5+* on spikelet fertility in BC_6_F_4_ families. **a** Spikelet fertility of homozygote (left) and hemizygote (right) of *ORF5+* transgene. **b** Mean spikelet fertility value of homozygotes (left) and hemizygotes (right) of *ORF5+* transgene. Data represent means ± SEM. Significant differences are indicated by **P < 0.01

To study the genetic effects of *qSIG3.1, qSIG3.2, qSIG6.1*, and *qSIG12.1*, advanced NILs for each locus were developed, containing corresponding chromosomal segments from DL in the background of BL (Table S2). In BC_6_F_4_ NILs, when all four loci *qSIG3.1*, *qSIG3.2*, *qSIG6.1* and *qSIG12.1* were DL/DL homozygous, the SF of *ORF5+* hemizygotes and homozygotes were 61.70 ± 1.00% and 17.47 ± 1.37% respectively (Fig. 3). The results suggested that the *ORF5+* copy number greatly affected SF and only plants with hemizygous transgenic *ORF5+* should be appropriate for conducting genetic effects analysis of *qSIG3.1*, *qSIG3.2*, *qSIG6.1*, and *qSIG12.1*. When the genetic effect of individual QTL was analyzed, not only should the other three QTLs be DL homozygous (DL/DL), but also transgenic *ORF5+* should be hemizygous.

For *qSIG3.1*-NIL, the SF of plants with genotypes *qSIG3.1*-BL/BL, *qSIG3.1*-BL/DL and *qSIG3.1*-DL/DL were 20.76 ± 0.76%, 59.57 ± 0.73% and 61.70 ± 1.00% respectively (Table 4). There were significant difference in SF between *qSIG3.1*-BL/BL and *qSIG3.1*-BL/DL, but no apparent SF difference between *qSIG3.1*-BL/DL and *qSIG3.1*-DL/DL. These results suggest that *qSIG3.1* plays a significant role in SF, with the DL allele displaying complete dominance.

**Table 4:** Spikelet fertility of different genotypes in *qSIG3.1*-NIL, *qSIG3.2*-NIL, *qSIG6.1*-NIL, and *qSIG12.1*-NIL

QTL	Generation	Genotype	Number of plants	SF (%) ^#^	*P* value
*qSIG3.1*	BC_6_F_4_	BL/BL	24	20.76 ± 0.76	0.000^a^, 0.083^b^, 0.000^c^
		BL/DL	32	59.57 ± 0.73	
		DL/DL	24	61.70 ± 1.00	
*qSIG3.2*	BC_6_F_4_	BL/BL	32	38.76 ± 1.22	0.000^a^, 0.000^b^, 0.000^c^
		BL/DL	64	49.72 ± 0.89	
		DL/DL	30	56.76 ± 1.01	
*qSIG6.1*	BC_6_F_4_	BL/BL	18	45.46 ± 1.70	0.050^a^, 0.028^b^, 0.000^c^
		BL/DL	31	50.21 ± 1.50	
		DL/DL	13	56.11 ± 1.77	
*qSIG12.1*	BC_4_F_6_	BL/BL	9	38.31 ± 2.56	0.000^a^, 0.000^b^, 0.000^c^
		BL/DL	22	59.50 ± 1.66	
		DL/DL	9	70.60 ± 1.05	

*SF* spikelet fertility, *BL/DL* heterozygous Balilla/Dular, *DL/DL* homozygous Dular/Dular; ^a^probability obtained from a *t*-test of BL/BL genotype against BL/DL genotype within the same family; ^b^probability obtained from a *t*-test of BL/DL genotype against DL/DL genotype within the same family; ^c^probability obtained from a *t*-test of BL/BL genotype against DL/DL genotype within the same family. ^#^means ± SEM

The SF of *qSIG3.2*-NIL plants with genotypes *qSIG3.2*-BL/BL, *qSIG3.2*-BL/DL and *qSIG3.2*-DL/DL were 38.76 ± 1.22%, 49.72 ± 0.89% and 56.76 ± 1.01% respectively (Table 4). The difference in SF between plants with different genotypes was significant. Nevertheless, the SF of plants with heterozygous genotype *qSIG3.2*-BL/DL fell between that of *qSIG3.2*-BL/BL and *qSIG3.2*-DL/DL, indicating that the DL allele at *qSIG3.2* was incompletely dominant.

The SF of *qSIG6.1*-NIL plants with genotypes *qSIG6.1*-BL/BL, *qSIG6.1*-BL/DL and *qSIG6.1*-DL/DL were 45.46 ± 1.70%, 50.21 ± 1.50% and 56.11 ± 1.77% respectively (Table 4). There were minor differences in SF between each genotype. The SF of plants with the heterozygous genotype *qSIG6.1*-BL/DL also fell between that of *qSIG6.1*-BL/BL and *qSIG6.1*-DL/DL, indicating that the DL allele at *qSIG6.1* displayed partial dominance.

The SF of *qSIG12.1*-NIL plants with genotypes *qSIG12.1*-BL/BL, *qSIG12.1*-BL/DL and *qSIG12.1*-DL/DL were 38.31 ± 2.56%, 59.50 ± 1.66% and 70.60 ± 1.05% respectively (Table 4). The SF of plants was significantly different between two different genotypes. The SF of plants with the heterozygous genotype *qSIG12.1*-BL/DL also fell between that of *qSIG12.1*-BL/BL and *qSIG12.1*-DL/DL, demonstrating that the DL allele at *qSIG12.1* had incomplete dominance*.*

## Discussion

Inter-specific or Inter-subspecific HS is caused by postzygotic reproductive isolation, which limits gene flow between species or subspecies. Therefore, HS genes are also known as speciation genes (Bateson 1909; Dobzhansky 1937; Muller 1942). The study of these genes in rice could help provide insight into the origin and differentiation of rice subspecies (Du et al. 2011; Ouyang et al. 2016).

Previous studies showed that the *S5* locus on chromosome 6 originated from the Oryzeae tribe, most likely through *Helitron* transposition (Ouyang et al. 2016). The ancestral genotype of the three genes of the *S5* locus is *ORF3+*/*ORF4+*/*ORF5+*, which mutated into *ORF3+*/*ORF4−*/*ORF5+* and *ORF3+*/*ORF4+*/*ORF5-*. Finally, a trigenic reproductive isolation system was formed between *indica* and *japonica* rice (Ouyang et al. 2016). However, other genes are involved in the reproductive isolation caused by *S5*. The study of both *S5* and its interacting genes will deepen our understanding of the evolutionary mechanism of rice.

It has been suggested that *ORF4+* and *ORF5+* together lead to an endoplasmic reticulum stress response, while *ORF3+* prevents the stress response (Yang et al. 2012; Zhu et al. 2017). However, the detailed molecular mechanism remains elusive. For example, it is not known what are the targets of *ORF5+*, how stress response is transmitted, or at which stage *ORF3+* prevents a stress response. The study of SIG might fill this gap. Additionally, the results would also provide a valuable reference to the study of the molecular mechanism of other HS.

The cooperation between *ORF5+* (killer) and *ORF4+* (partner) of *S5* results in the abortion of unguarded female gametes (carrying *ORF3-*), while female gametes carrying *ORF3+* (protector) survive (Yang et al. 2012). The HS effect of *S5* in the background of *japonica* rice was greater than that of *indica* rice (Yang 2012). The suicidal *S5* haplotype has not been found in natural varieties (Yang et al. 2012). Therefore, the *ORF5+* allele was transformed into *japonica* rice BL to form BL^*ORF5+*^ with suicidal *S5* genotype (*ORF3-*, *ORF4+*, and *ORF5+*).

Although the SF of hybrid F_1_ DL/BL^*ORF5+*^ was as high as 65% (Fig. 1), the plants heterozygous for *qSIG3.1*, *qSIG3.2*, *qSIG6.1* and *qSIG12.1* were sterile in the BC_8_F_1_ generation, with an SF of only 15.93% (Table S4). This difference in SF between F_1_ and BC_8_F_1_ probably originates from the difference in copy number of *ORF4+.* DL/BL^*ORF5+*^ has a single copy of *ORF4+* while BC_8_F_1_ has two copies of *ORF4+*. Yang et al. (2012) found a considerable dosage effect for both *ORF4+* and *ORF5+*, without the presence of *ORF3+*. Both DL (*ORF3-*, *ORF4-*, and *ORF5n*) and BL (*ORF3-*, *ORF4+*, and *ORF5-*) carry the *ORF3-* allele, so all mapping populations used in the present research carry homozygous *ORF3-*, and the effect of *S5* on SF was mainly dependent on copy numbers of *ORF4+* and *ORF5+*. Similarly, since the copy number of *ORF5+* greatly affected SF, it was preferable to keep hemizygous transgenic *ORF5+* for genetic effect analysis (Fig. 3).

Four QTL *qSIG3.1, qSIG3.2, qSIG6.1*, and *qSIG12.1*, were mapped in the present study. The genetic effect of the DL alleles differed among them: partially dominant at *qSIG3.2, qSIG6.1*, and *qSIG12.1*, while completely dominant at *qSIG3.1*. Of the QTL identified, *qSIG3.1* displayed the greatest genetic effect, and is the most potential for gene cloning. Indeed, the DL allele could improve SF by approximately 40% in the BC_6_F_4_ *qSIG3.1-*NIL. We have constructed a fine-mapping population and expect to clone *qSIG3.1* in the near future.

## Conclusions

Four *S5*-interacting QTL, *qSIG3.1*, *qSIG3.2*, *qSIG6.1* and *qSIG12.1*, were identified by genetic mapping. The effects of each QTL were analyzed using advanced backcross NIL. Of these, *qSIG3.1* with potential breeding value exhibited the greatest genetic effect. The DL allele of *qSIG3.1* is completely dominant. The effect of the other three loci was relatively small and their DL alleles were found to be partially dominant. The results of the present study would have laid the groundwork for the elucidation of the molecular mechanism of HS caused by *S5* in rice.

## Supplementary Information

**Additional file 1: Figure S1.** Detection of *ORF5+* transgenic plants. **Figure S2.** Genetic linkage map of the F_2_ population derived from the cross between Dular and Balilla^*ORF5+*^. **Figure S3.** Additional mapping of *qSIG3.1*. **Figure S4** Genotype of BC_3_F_3_ individuals with high SF. **Figure S5.** Rice genome 6 K-microarray analysis of 18MR47–17 and 18MR47–19. **Table S1.** Detailed information of primers. **Table S2.** Detailed information of NILs for each locus. **Table S3.** Genotypes of 173 F_2_ individual plants for two flank markers of *qSIG5.1*. **Table S4.** SF and genotypes of plants in different generations.

## Data Availability

All datasets generated for this study are included in the manuscript/Additional Files.

## Abbreviations

HS: Hybrid sterility
SIG: *S5*-interacting gene
DL: Dular
BL: Balilla
SF: Spikelet fertility
NIL: Near isogenic lines
QTL: Quantitative trait loci
WCVs: Wide compatibility varieties
WCG: Wide compatibility gene
SD: Standard deviations
SEM: Standard error of mean
CTAB: Cetyl trimethyl ammonium bromide
GUS: *β*-glucuronidase
InDel: Insert and deletion
SARF: Sum of adjacent recommendation frequencies
ICIM: Inclusive composite interval mapping
PCR: Polymerase chain reaction
SNP: Single nucleotide polymorphism
LOD: Log odds score
PVE: Phenotypic variation explained by the marker
Add: Additive effect
Dom: Dominance effect
