# Supplementary material for: Genetic Analysis of S5-Interacting Genes Regulating Hybrid Sterility in Rice
Source: Rice (N Y). 2021 Jan 9;14:11. doi: 10.1186/s12284-020-00452-x (PMC7797014; doi:10.1186/s12284-020-00452-x)
Supplement: Supplementary file 1 — Additional file 1: Figure S1. Detection of ORF5+ transgenic plants. Figure S2. Genetic linkage map of the F2 population derived from the cross between Dular and BalillaORF5+. Figure S3. Additional mapping of qSIG3.1. Figure S4 Genotype of BC3F3 individuals with high SF. Figure S5. Rice genome 6 K-microarray analysis of 18MR47–17 and 18MR47–19. Table S1. Detailed information of primers. Table S2. Detailed information of NILs for each locus. Table S3. Genotypes of 173 F2 individual plants for two flank markers of qSIG5.1. Table S4. SF and genotypes of plants in different generations. [file 12284_2020_452_MOESM1_ESM.doc]

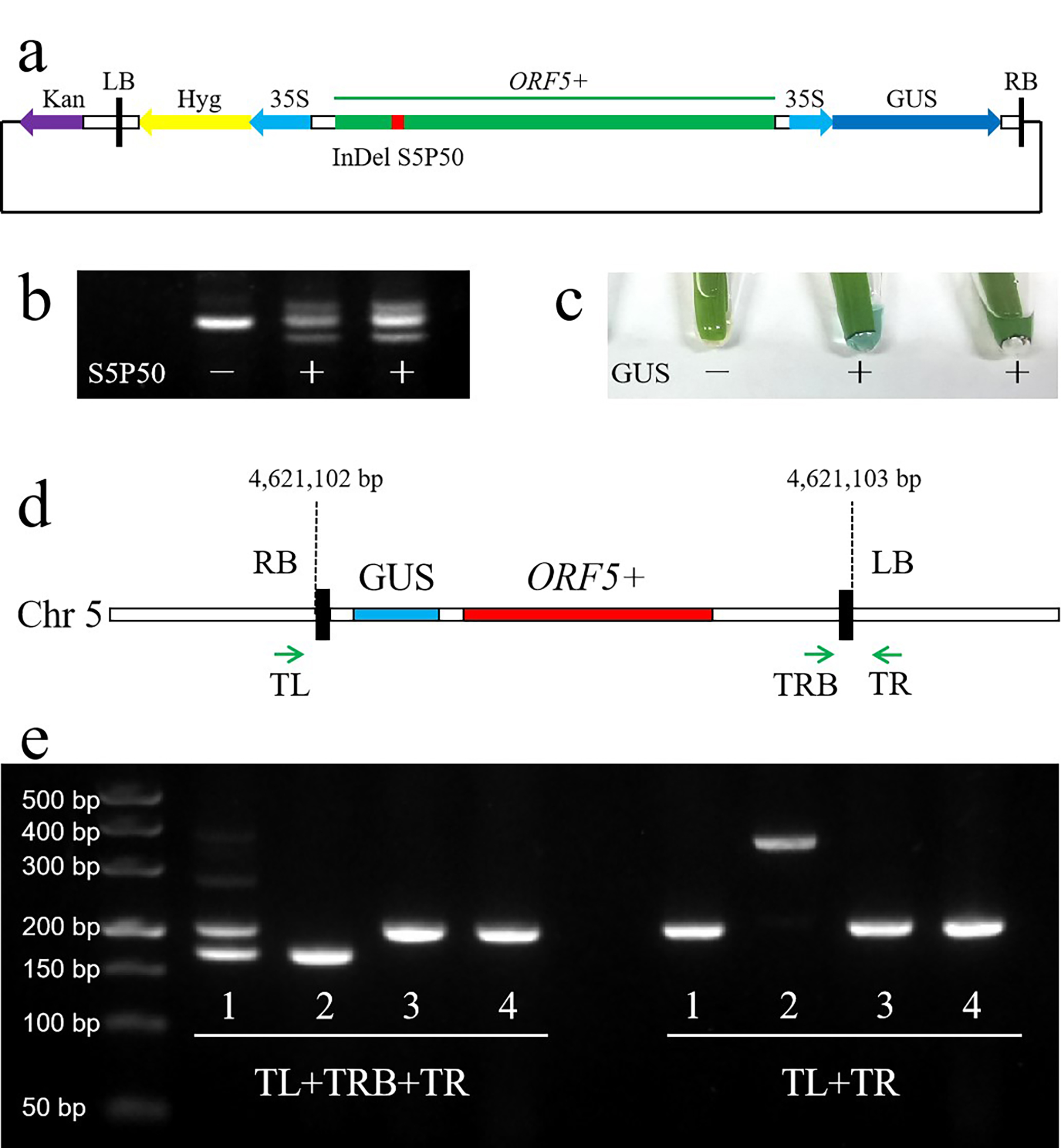


**Figure S1** Detection of *ORF5+* transgenic plants. (**a**) The diagram of vector for *ORF5+* transformation. RB, right border; LB, left border. **(b)** Detection of InDel marker S5P50. +, transgenic positive plants; -, transgenic negative plants. **(c)** GUS staining of transgenic plants. +, transgenic positive plants; -, transgenic negative plants. **(d)** Designed primers for the detection of insertion of transgenic *ORF5+*. (**e**) PCR amplification with primer TL, TRB, and TR. Lane 1, hemizygote of *ORF5+* transgene; Lane 2, homozygote of *ORF5+* transgene; Lane 3, Balilla; Lane 4, Dular.


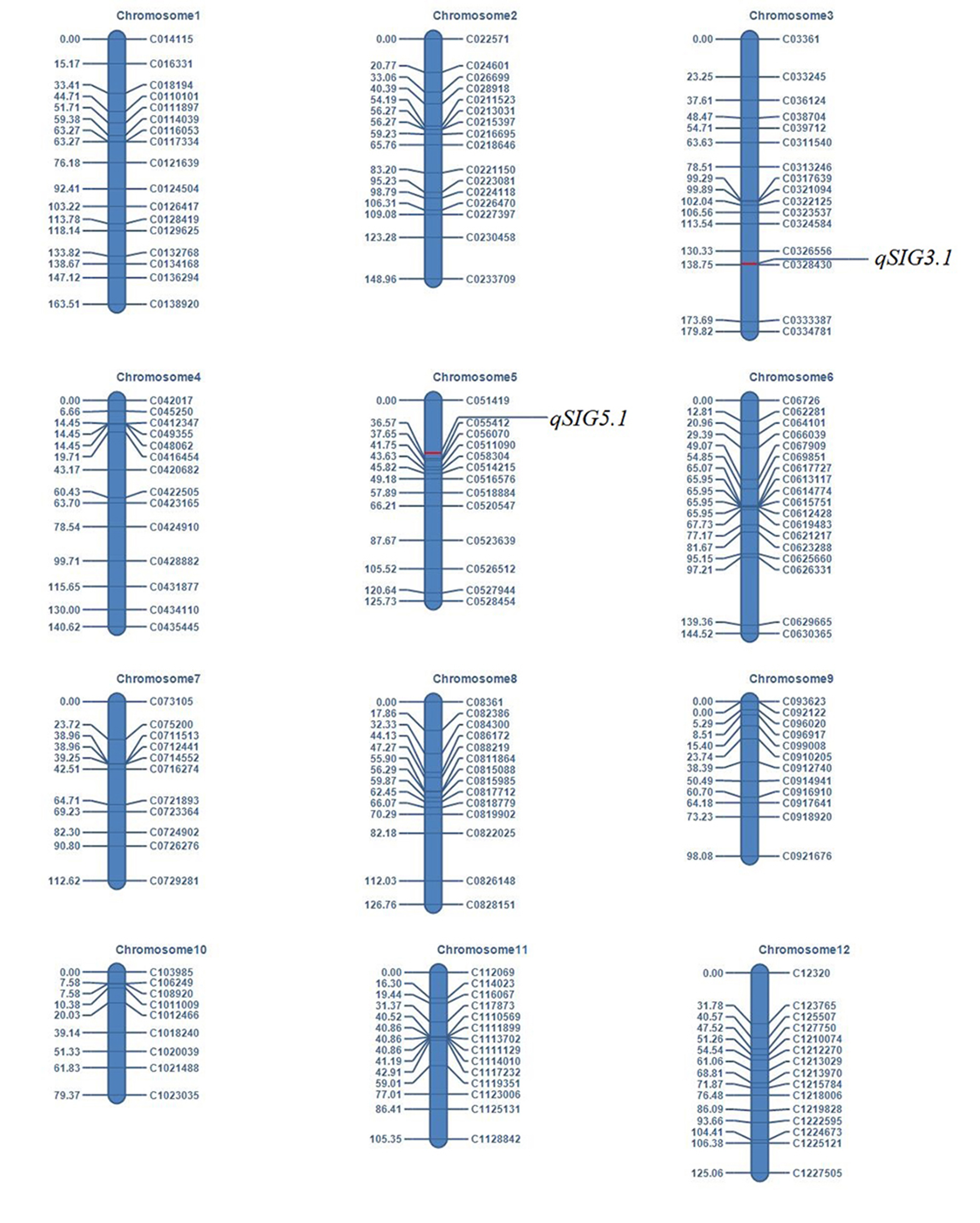


**Figure S2** Genetic linkage map of the F_2_ population derived from the cross between Dular and Balilla*^ORF5+^*. *qSIG3.1* was located between marker C0326556 and C0328430, while *qSIG5.1* was located between C051419 and C055412.


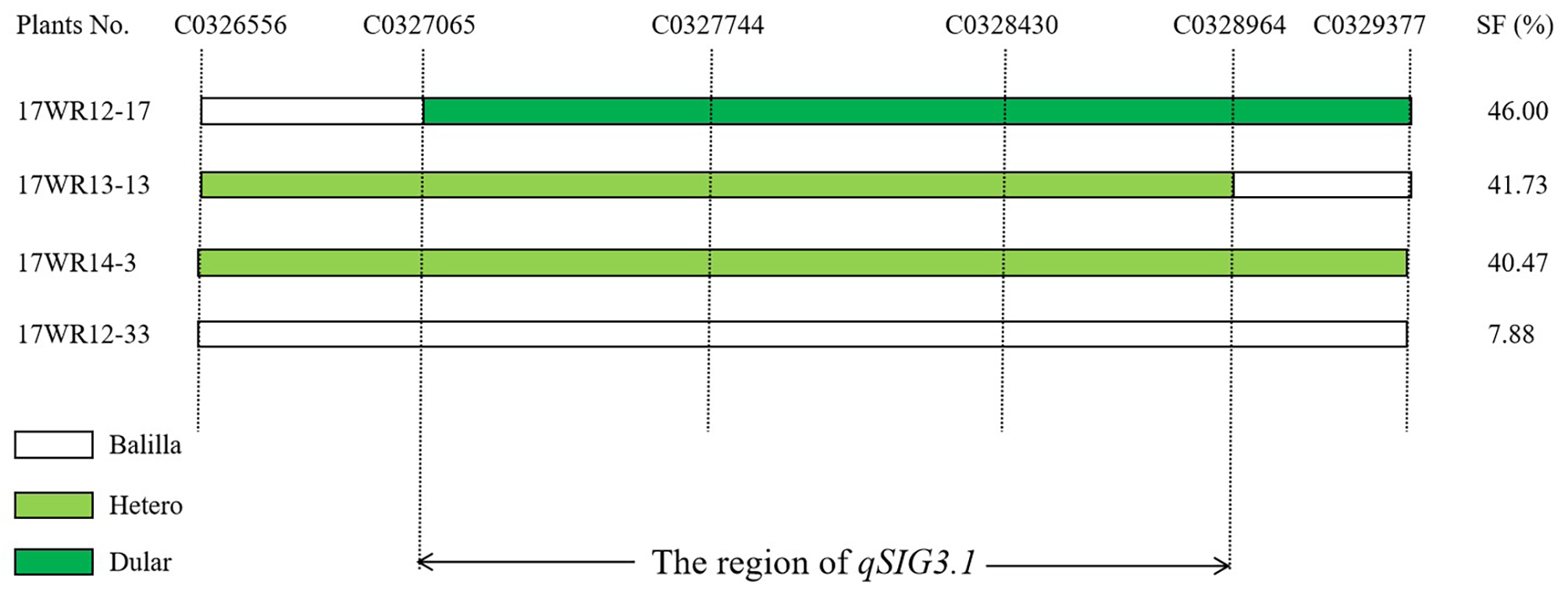


**Figure S3** Additional mapping of *qSIG3.1*. The location of *qSIG3.1* was narrowed down to between markers C0327065 and C0328964 using several recombinants in BC_4_F_3_. SF, spikelet fertility.


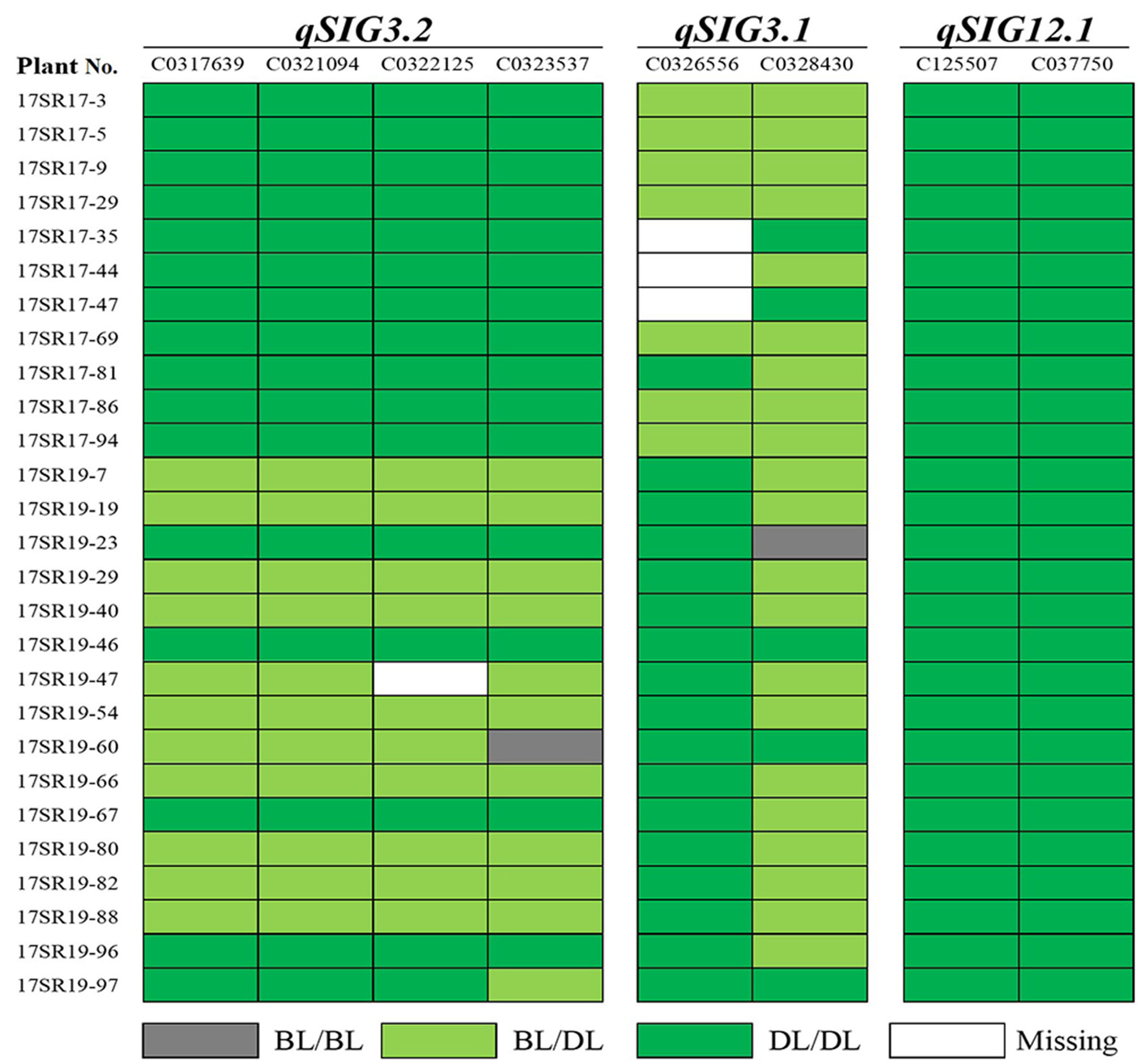


**Figure S4** Genotype of BC_3_F_3_ individuals with high SF. BL/BL, homozygous Balilla/Balilla; BL/DL, heterozygous Balilla/Dular; DL/DL, homozygous Dular/Dular; Missing, missed data.


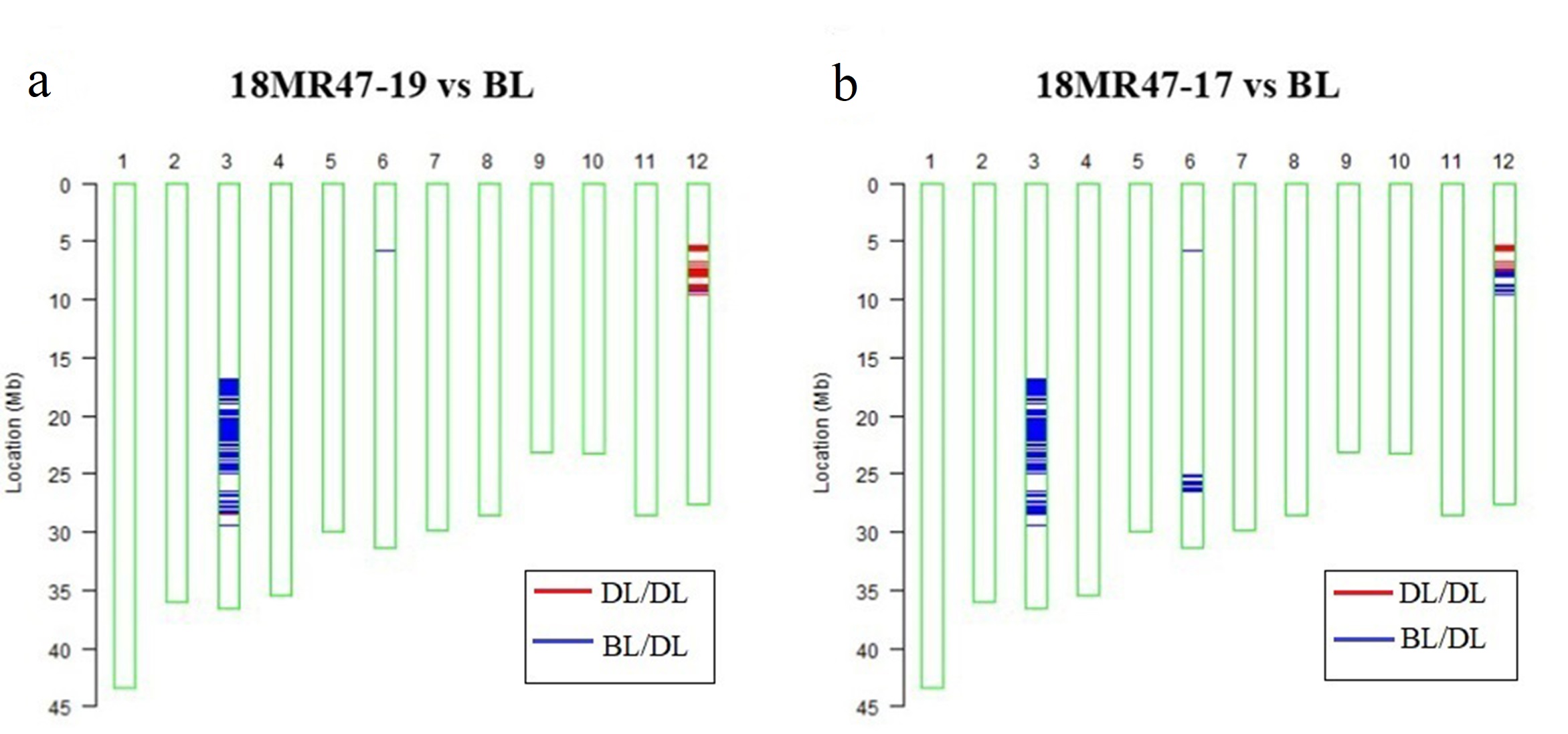


**Figure S5** Rice genome 6K-microarray analysis of 18MR47-19 (**a**) and 18MR47-17 (**b**). Both results were based on the comparison with their recurrent parent BL. Since the *qSIG5.1* is in fact caused by insertion of transgenic *ORF5+*, and the probes on the microarray are fixed according to the position of the chromosomes. So, the fragment of transgenic *ORF5+* will only bind to the probes of *ORF5-* on chromosome 6 of the microarray. BL/DL, heterozygous Balilla/Dular; DL/DL, homozygous Dular/Dular.

**Table S1** Detailed information of primers.

| Pimer Name | Sequence (5’-3’) |
| --- | --- |
| S5P50F | GAACACCTCGAATAAGCT |
| S5P50R | CTGCTGCCTCTGTGTCTA |
| C0327065F | AGTATTTTAGATGTGAACCCTT |
| C0327065R | GAACACGACTGTAACTCCCT |
| C0328964F | TTGCCAAGTTCCGATCACAG |
| C0328964R | CCAGGAATCGCTTAATACGG |
| C0317639F | GGTTCCGAAAATCGAACATTTCA |
| C0317639R | GGCTGTGTTTAAGTTCAGTGC |
| C0323537F | GATTCCTCTTTAGTCATTTCAGTTG |
| C0323537R | TATAAGGATTTCTACACTGATTCCC |
| C0625165F | AAAGTCATTTAAGGAATGAATAGAG |
| C0625165R | GATAAGCCCGGAACTGGTA |
| C0626545F | ATTTGGATCACGGGATTAT |
| C0626545R | GATTCGATGTTTTGTTTTCTATG |
| C124702F | CACGTACAGTGGCCGTGGAA |
| C124702R | CCAAATCACGTCGCCGCATT |
| C125855F | GATCATACAGCTCTTAGCGG |
| C125855R | CTGCAAACTTCACAACATAAAT |
| TL | TCAGCCATAGCCATTGAGTT |
| TRB | GTGTTATTAAGTTGTCTAAGCGTC |
| TR | ATCGGATACTGGCTCGTATA |
| ULB2 | CCAGATAAGGGAATTAGGGTT |
| XRB1 | GTGAATTACAGGTGACCAGCTC |
| pCM13-L | CAGGAAACAGCTATGAC |
| pCM13-R | TGTAAAACGACGGCCAGT |

**Table S2** Detailed information of NILs for each locus.

| Generation | NIL Name | QTL Genotype | | | | *ORF5+* |
| --- | --- | --- | --- | --- | --- | --- |
|  |  | *qSIG3.1* | *qSIG3.2* | *qSIG6.1* | *qSIG12.1* |  |
| BC_6_F_4_ | *qSIG3.1*-NIL | BL/DL | DL/DL | DL/DL | DL/DL | Hemizygote |
| BC_6_F_4_ | *qSIG3.2*-NIL | DL/DL | BL/DL | DL/DL | DL/DL | Hemizygote |
| BC_6_F_4_ | *qSIG6.1*-NIL | DL/DL | DL/DL | BL/DL | DL/DL | Hemizygote |
| BC_4_F_6_ | *qSIG12.1*-NIL | DL/DL | DL/DL | DL/DL | BL/DL | Hemizygote |

NIL, near isogenic line; QTL, quantitative trait loci; BL/DL, heterozygous Balilla/Dular; DL/DL, homozygous Dular/Dular.

**Table S3** GenotypeS of 173 F_2_ individual plants for two flank markers of *qSIG5.1*.

| Marker Name | Genotype | Number of plants | Chi-Square (1:2:1) | Pr>ChiSq |
| --- | --- | --- | --- | --- |
| C051419 | BL/BL | 53 | 11.5263 | 0.0031 |
|  | BL/DL | 94 |  |  |
|  | DL/DL | 24 |  |  |
|  | Missing | 2 |  |  |
| C055412 | BL/BL | 56 | 51.1302 | 0.0000 |
|  | BL/DL | 111 |  |  |
|  | DL/DL | 2 |  |  |
|  | Missing | 4 |  |  |

BL/DL, heterozygous Balilla/Dular; DL/DL, homozygous Dular/Dular.

**Table S4** The SF and genotype of plants in different generations.

| Generation | QTL Genotype | | | | *ORF5+* | SF (%) ^#^ |
| --- | --- | --- | --- | --- | --- | --- |
|  | *qSIG3.1* | *qSIG3.2* | *qSIG6.1* | *qSIG12.1* |  |  |
| F_1_*^ORF5+^* | BL/DL | BL/DL | BL/DL | BL/DL | Hemizygote | 65.00 ± 1.36 |
| BC_8_F_1_ | BL/DL | BL/DL | BL/DL | BL/DL | Hemizygote | 15.93 ± 1.32 |

SF, spikelet fertility; QTL, quantitative trait loci; BL/DL, heterozygous Balilla/Dular; DL/DL, homozygous Dular/Dular. ^#^means ± SEM.
